# An interpretable AutoML-based prediction model for enteral nutrition intolerance in severe pulmonary tuberculosis patients and development of a clinical decision system

**DOI:** 10.3389/fnut.2026.1816436

**Published:** 2026-06-22

**Authors:** Zhen Li, Qingfeng Wu, Xiaoxia Qi, Lei Pan, Jing Xu

**Affiliations:** 1 Hangzhou Red Cross Hospital, Hangzhou, Zhejiang, China; 2 Qingchun Hospital, Hangzhou, Zhejiang, China

**Keywords:** clinical decision system, Divine Religions Algorithm, enteral nutrition, enteral nutrition intolerance, machine learning, severe pulmonary tuberculosis

## Abstract

**Objective:**

This study aims to construct an AutoML-based predictive model for enteral nutrition intolerance (ENI) in severe pulmonary tuberculosis (PTB) patients and develop a visualized clinical decision support system to inform personalized nutrition management.

**Methods:**

Using a multicenter retrospective cohort design, clinical data from 645 severe PTB patients were analyzed. An Improved Dimension-wise Gaussian-mutated Chaotic Divine Religions Algorithm (IDRA) was proposed and integrated into the AutoML framework to simultaneously optimize feature selection and hyperparameter tuning. SHapley Additive exPlanations (SHAP) were employed for both global and individual-level model interpretability, including waterfall plots, force plots, and dependence plots. A visualized clinical decision system was subsequently developed based on the optimal model.

**Results:**

(1) IDRA demonstrated superior convergence speed and lower local optima entrapment risk on CEC2022 benchmark functions; (2) Eight key predictors were identified and ranked by SHAP importance: hypoalbuminemia, anti-TB drug regimen (rifampicin-containing or not), formula type (short peptide vs. intact protein), age, BMI, EN initiation time, tube type (nasoenteric vs. nasogastric), and Chinese herbal application; (3) The optimal AutoML model achieved an AUC of 0.910 in internal validation and 0.880 in external validation; (4) Individual-level SHAP analysis of representative high-, medium-, and low-risk cases using waterfall plots and force plots demonstrated clinically coherent differential model outputs under varying feature combinations, corroborating global interpretation results.

**Conclusion:**

This study established an interpretable AutoML-based prediction model and clinical decision system for enteral nutrition intolerance in severe PTB patients. Its core innovation lies in creating a transparent, user-friendly, and high-efficacy precision risk-assessment paradigm that provides both population-level feature attribution and case-level decision logic visualization, offering a novel approach to individualized EN tolerance prediction.

## 1 Introduction

Tuberculosis remains a major challenge in global public health. According to World Health Organization data, there are over 10 million new tuberculosis cases annually, with severe pulmonary tuberculosis patients accounting for approximately 12% of these cases (1). Due to disease severity, patients with severe pulmonary tuberculosis often experience enteral nutrition intolerance. This ultimately results in inadequate nutrient intake and reduced anabolic metabolism. Simultaneously, *Mycobacterium tuberculosis* consumes body proteins during metabolic processes, rendering patients highly susceptible to protein-energy malnutrition (2). Clinical statistics indicate that malnutrition affects up to 83.6% of severe pulmonary tuberculosis patients, which further compromises immune function and exacerbates pulmonary infection progression and mortality risk (3). Enteral nutrition (EN), as the preferred nutritional support strategy for critically ill patients, offers physiological digestive absorption alignment, intestinal mucosal barrier protection, and reduced infectious complications. It has been recommended as the core nutritional intervention for severe pulmonary tuberculosis patients in the “Guidelines for the Diagnosis and Treatment of Severe Pulmonary Tuberculosis (2023 Edition)” (4). However, due to unique pathophysiological conditions, these patients exhibit significantly higher rates of enteral nutrition intolerance during EN implementation compared to general critically ill patients. Studies show that gastrointestinal adverse reactions—including abdominal distension, diarrhea, gastric retention, and reflux aspiration—not only interrupt nutritional supply but may also trigger severe complications such as aspiration pneumonia and enterogenic infections. These issues may even necessitate discontinuing EN in favor of more invasive parenteral nutrition, increasing risks of liver injury and catheter-related infections (5, 6).

Early identification and intervention of enteral nutrition intolerance are critical for improving EN tolerance and nutritional support efficacy in severe pulmonary tuberculosis patients (7). Current clinical practice relies heavily on subjective assessments for evaluating enteral nutrition tolerance—such as physicians judging based on abdominal distension severity and defecation frequency—lacking objective, quantitative indicators. Some studies have attempted to implement gastrointestinal function scores, but these systems are primarily developed for general critically ill populations without adequate consideration of disease-specific factors in severe pulmonary tuberculosis. These include chronic hypoxia from tuberculosis, prolonged gastrointestinal mucosal irritation by anti-tuberculosis drugs, and gastrointestinal motility suppression by hypercapnia, thereby failing to meet clinical early-warning needs for enteral nutrition intolerance (8, 9).

With the application of precision medicine and artificial intelligence in critical care medicine, predictive modeling based on clinical big data has become crucial for disease risk assessment (10). Current research on EN-related gastrointestinal function prediction models focuses primarily on severe pancreatitis, stroke, and sepsis, with specialized studies for severe pulmonary tuberculosis remaining absent. Existing models predominantly incorporate conventional predictors such as age, APACHE II scores, and mechanical ventilation status, omitting tuberculosis-specific parameters including detailed nutritional protocols, specific biomarkers, and tuberculosis-related treatment factors. This limitation compromises model applicability for severe pulmonary tuberculosis populations (11). Concurrently, clinical decision systems serve as vital platforms for translating complex model calculations into intuitive clinical recommendations, aiding healthcare providers in rapid intervention planning (12). However, existing EN decision systems primarily emphasize formula selection and feeding rate adjustments, lacking dynamic prediction modules for enteral nutrition intolerance and corresponding intervention suggestions. Consequently, they fail to provide comprehensive “risk prediction of intolerance – intervention decision – effect feedback” support for severe pulmonary tuberculosis patients (13).

To address these gaps, this study focuses on severe pulmonary tuberculosis patients. Through retrospective analysis of clinical data, we aim to identify key risk factors for enteral nutrition intolerance, apply LASSO regression and AutoML for feature selection and model construction, systematically compare and evaluate the performance of multiple supervised machine learning models, validate model interpretability via SHAP analysis, and subsequently develop a visual clinical decision system to achieve early prediction and precision intervention for enteral nutrition intolerance in this population.

## 2 Materials and methods

### 2.1 Study design and participants

**Figure 1:**
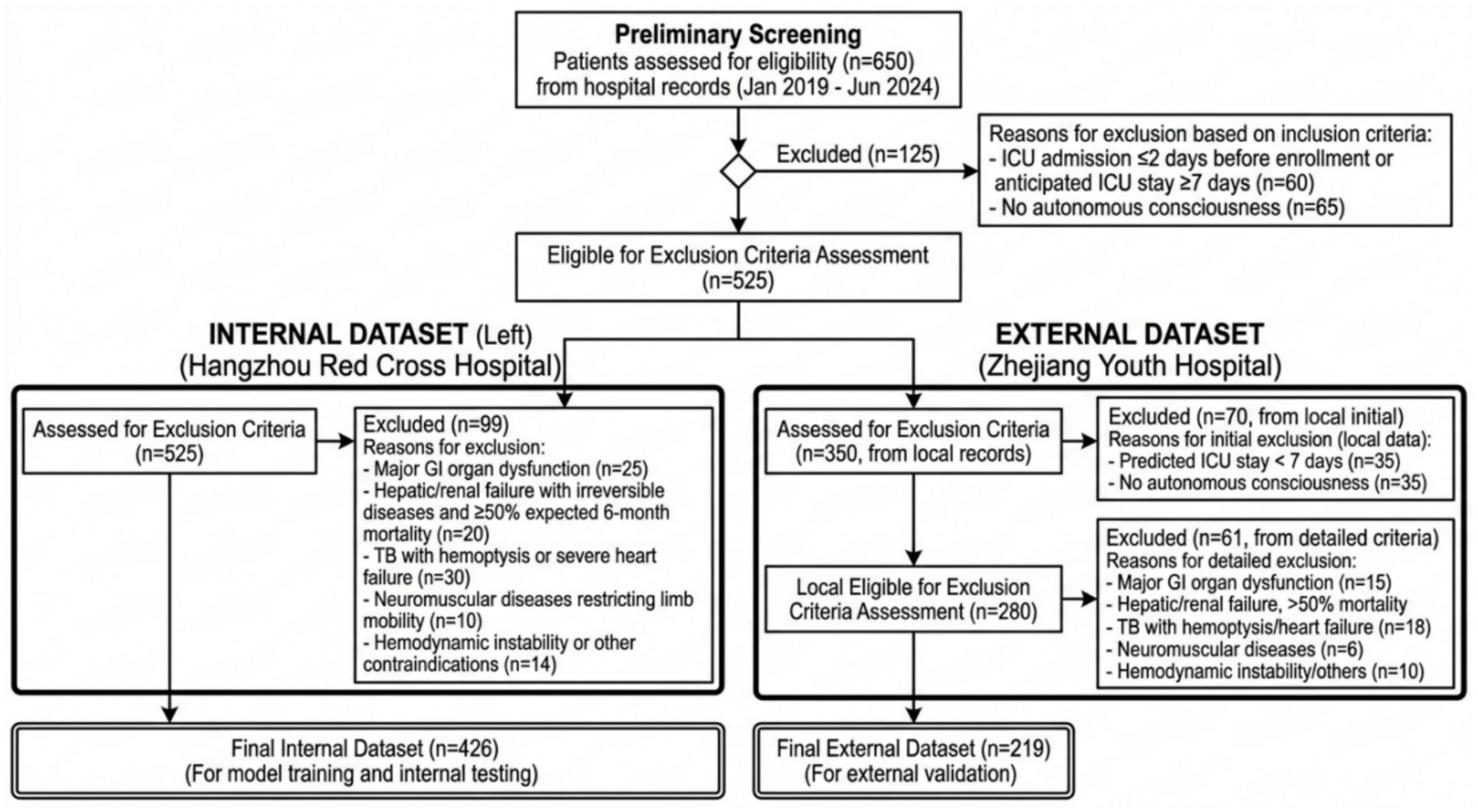
CONSORT flow diagram for study selection and analysis.

Our study adopted a multicenter retrospective cohort study design, collecting data from 426 patients with severe pulmonary tuberculosis admitted to our hospital (Hangzhou Red Cross Hospital) between January 2019 and June 2024 as the internal dataset for prediction model training and internal testing, while data from 219 patients with severe pulmonary tuberculosis admitted to another hospital (Zhejiang Youth Hospital) were collected to form an external test dataset for external validation of the model. Ethical approval for this study was obtained from the hospital ethics committee (approval number: 2025-205-001), and informed consent was waived due to the retrospective nature of the study. The sample screening process is shown in Figure 1.

Inclusion criteria: (1) Diagnosis of pulmonary tuberculosis per WS288—2017 Diagnostic Criteria for Pulmonary Tuberculosis and admission to tuberculosis ICU; (2) ICU admission ≤2 days before enrollment with anticipated ICU stay ≥7 days; (3) Adequate level of consciousness to communicate discomfort or symptoms of intolerance, defined as a Glasgow Coma Scale (GCS) score of 15 without continuous intravenous sedation, or a Richmond Agitation–Sedation Scale (RASS) score between 0 and −2 if light sedation was required during mechanical ventilation.

Exclusion criteria: (1) Major gastrointestinal organ dysfunction; (2) Hepatic/renal failure with irreversible diseases and ≥50% expected 6-month mortality; (3) Pulmonary tuberculosis with hemoptysis or severe heart failure; (4) Neuromuscular diseases restricting limb mobility (e.g., myasthenia gravis, amyotrophic lateral sclerosis); (5) Hemodynamic instability, deep vein thrombosis, fractures requiring immobilization, or bleeding tendency.

Data were collected using standardized electronic forms and independently verified by two clinicians. Variables included: (1) Baseline characteristics: Age, sex, BMI, albumin (ALB), C-reactive protein (CRP) levels; (2) Tuberculosis severity: Number of lung cavities on high-resolution CT, sputum smear positivity rate (%); (3) Comorbidities: Diabetes (diagnosed per ADA criteria), hypoalbuminemia (3.0 g/dL) (14); (4) Treatment factors: Anti-TB regimens with high gastrointestinal toxicity; (5) Nutritional protocol: Tube type (nasogastric/nasoenteric), progressive early mobilization (yes/no), Chinese herbal acupoint application (yes/no), formula type (intact protein/short peptide), caloric target (kcal), EN initiation time (hours post-admission). The outcome indicator was enteral nutrition intolerance (ENI), defined as follows: during the implementation of enteral nutrition (EN), if gastrointestinal symptoms such as abdominal distension, vomiting, diarrhea, and increased gastric residual volume (GRV) occur, ultimately leading to the interruption of enteral nutrition support or nutritional intake failing to reach 80% of the support target, and after excluding contraindications such as complete intestinal obstruction, ENI can be diagnosed (15).

The data completeness was good, with the missing proportion of all variables being less than 1%. Missing values for continuous variables were imputed using the mean, and missing values for categorical variables were imputed using the mode. All imputations were performed independently within the training set, internal test set, and external test set to avoid information leakage.

### 2.2 Automated machine learning model construction

To construct a robust prognostic prediction model without information leakage, this study designed and implemented a rigorous double-layer nested cross-validation automated machine learning (AutoML) framework, with the Improved Divine Religions Algorithm (IDRA) as its core. The pseudocode of the model framework is provided in Supplementary Material 1.

#### 2.2.1 Improved Divine Religions Algorithm

The base algorithm selected for this study was the Divine Religions Algorithm (DRA) (16); however, it has issues such as a tendency to fall into local optima. Building upon this algorithm, this study proposes an Improved Divine Religions Algorithm (IDRA) based on dimension-wise Gaussian mutation and chaos. This algorithm first utilizes the Cubic chaotic mapping strategy to initialize the population, overcoming the blindness of population initialization. Subsequently, dimension-wise Gaussian mutation is applied to the optimal individual to enhance its ability to escape local optima and improve the global search capability of the algorithm. To validate the optimization capability of the improved IDRA algorithm, comparative tests were conducted against the original DRA, Whale Optimization Algorithm (WOA), Grey Wolf Optimizer (GWO), Particle Swarm Optimization (PSO), Genetic Algorithm (GA), Genetic Algorithm-Particle Swarm Optimization (GA-PSO) and Genetic Algorithm-Ant Colony Optimization (GA-ACO) algorithms. Experiments employed all 12 benchmark functions from the CEC2022 test set, with variable dimensions set to 10, population size to 30, and maximum iterations to 500. Each algorithm was independently run 30 times to ensure statistical reliability. Box plots were generated to evaluate optimization stability based on 30-run results.

#### 2.2.2 Nested cross-validation modeling process

The entire modeling process was designed to strictly isolate the model development phase from the final evaluation phase, ensuring that the test set remained completely unused before the model was finalized. The specific steps were as follows: (1) Data splitting: The total dataset was randomly divided into a one-time locked independent internal test set (*n* = 85) and a training and development set (*n* = 341) at an 8:2 ratio. This test set remained isolated throughout all subsequent model optimization and selection steps and was used solely for the final model performance evaluation. (2) Outer loop: Five-fold cross-validation was adopted as the outer loop on the training and development set. In each fold iteration, the data were further divided into a “training fold” and a “validation fold.” (3) Inner loop: On the “training fold” of each outer iteration, IDRA-driven AutoML inner optimization was executed. IDRA performed a dual task at this stage: (a) feature subset selection in the discrete space, screening out the most predictive combination from all candidate features; (b) hyperparameter tuning for the selected machine learning model in the continuous space. The optimization objective was to minimize the objective function calculated through cross-validation on the “training fold.” (4) Model training and validation: Using the optimal feature subset and hyperparameter configuration determined by the inner loop, the model was retrained on the complete “training fold,” and its performance was evaluated on the independent “validation fold.” The evaluation metrics for that fold were recorded. (5) Repetition and integration: Steps 2–4 were repeated to complete the five-fold outer loop. Ultimately, we obtained five sets of model configurations with optimal performance on their respective validation folds and their performance estimates. To obtain a stable final model, we used the consensus feature set screened during the five-fold optimization process and retrained an integrated AutoML model on the entire training and development set using the median of the five-fold hyperparameters. Final evaluation: The final integrated AutoML model, finalized on the complete training and development set in step 5, was applied to the completely independent internal test set and external test set to calculate all reported performance metrics.

Meanwhile, for comparison, we synchronously established five benchmark models: Logistic Regression (LR), Support Vector Machine (SVM), Adaptive Boosting (AdaBoost), Extreme Gradient Boosting (XGBoost), and Light Gradient Boosting Machine (LightGBM). The five benchmark models used the grid search method for hyperparameter optimization and LASSO for feature selection. All models were trained and compared under the same nested cross-validation framework and used standardized data.

### 2.3 Evaluation metrics

This study established a multi-dimensional evaluation system: (1) Classification performance: For the prognostic prediction model, six core metrics were adopted, including Accuracy (ACC), Sensitivity (SEN), Specificity (SPE), F1 score (the harmonic mean comprehensively considering precision and recall), Area Under the ROC Curve (AUC), and Area Under the Precision-Recall Curve (AUPRC), to systematically evaluate the model’s discriminative ability and stability under class imbalance scenarios; (2) Calibration performance: Calibration curves combined with the Brier score (where lower values indicate more accurate predictions) were used to assess the precision of probabilistic predictions. (3) Clinical application: Decision Curve Analysis (DCA) was applied to quantify the clinical utility of the model, calculating the Net Benefit (NB) at different threshold probabilities. By comparing NB with the reference lines of traditional intervention strategies, the effective range of the model in assisting decision-making was validated.

### 2.4 Interpretability analysis

After the preliminary screening of prognostic prediction features through the AutoML framework, this study further employed LASSO regression analysis to verify the robustness of the screened features, and finally utilized the SHAP interpretability model to elucidate the clinical rationality of the features. The specific process was as follows: (1) AutoML feature preliminary screening: Based on a predefined search space and optimization objective, the AutoML algorithm was utilized to automatically identify a feature subset significantly associated with prognosis; (2) LASSO feature verification: LASSO regression was applied to the feature subset screened by AutoML to verify its sparsity and stability through the regularization constraint mechanism, ensuring the key features’ resistance to overfitting; (3) SHAP (Shapley Additive Explanations) interpretability analysis: The interpretation of the model logic was strictly conducted based on the SHAP framework derived from coalitional game theory. By calculating Shapley values, numerical attribution of feature contributions to individual predictions was performed, and summary plots were comprehensively used to reveal the global feature importance ranking, while waterfall plots, decision path plots, and force plots were employed to intuitively present the explanation process of specific predictions.

### 2.5 Visualization system development

Based on the intolerance prediction model constructed in this study, we developed an offline-operable web-based clinical decision support tool using Python 3.7 for risk assessment and clinical communication. The system adopted a single-page web application architecture (HTML), completing data entry, logical computation, and result presentation locally in the browser, requiring no additional software installation and no reliance on external networks or servers. Users input key predictive variables into the interface, and the system instantly outputs a percentage estimate of intolerance risk, a risk stratification alert, and concise clinical management key points. To protect privacy and facilitate deployment, the tool does not collect, upload, or relay any patient data.

### 2.6 Statistical methods

All study data were uniformly imported into the SPSS 26.0 statistical analysis platform for standardized processing. Continuous variables were expressed as mean ± standard deviation, and categorical variables were expressed as frequency (percentage). Comparisons of baseline characteristics among the three datasets were performed using one-way analysis of variance (ANOVA, for continuous variables, reporting the *F* value) or the chi-square test (for categorical variables, reporting the *χ*^2^ value); non-normally distributed variables were analyzed using the Kruskal–Wallis test (reporting the *H* value). The test power was based on the *p*-value (two-sided test, with the significance threshold set at *α* = 0.05), and the study results were presented in a structured tabular format.

## 3 Results

### 3.1 Baseline characteristics

A total of 645 patients with severe pulmonary tuberculosis were included in this study. The mean age was 65.35 ± 9.93 years, with 385 males (59.7%) and 260 females (40.3%). The mean BMI was 20.24 ± 2.26 kg/m^2^, and the overall incidence of enteral nutrition intolerance was 39.8% (257/645). There were no statistically significant differences across all baseline characteristics among the training set (*n* = 341), internal test set (*n* = 85), and external test set (*n* = 219) (*p* > 0.05), indicating good comparability among the three datasets and appropriate data partitioning. The intolerance rates were 39.9% (136/341) in the training set, 40.0% (34/85) in the internal test set, and 39.7% (87/219) in the external test set, with no significant difference in outcome proportion among the three groups (*χ*^2^ = 0.002, *p* = 0.999). Detailed results of the statistical tests are presented in Table 1.

**Table 1:** Comparison of baseline characteristics of patients across datasets.

Feature	Training set (*n* = 341)	Internal test set (*n* = 85)	External test set (*n* = 219)	Statistic	*P*-value
Baseline characteristics					
Age (years), mean ± SD	65.20 ± 10.18	64.85 ± 8.80	65.79 ± 9.96	*F* = 0.357	0.700
Male, *n* (%)	212 (62.17%)	51 (60.00%)	122 (55.71%)	*χ*^2^ = 2.318	0.314
BMI (kg/m^2^), mean ± SD	20.11 ± 2.28	20.32 ± 2.10	20.41 ± 2.27	*F* = 1.283	0.278
Albumin (g/dL), mean ± SD	2.83 ± 0.45	2.78 ± 0.43	2.83 ± 0.41	*F* = 0.581	0.560
C-reactive protein (mg/L), median [IQR]	47.9 [34.4–64.5]	50.4 [35.7–70.8]	49.3 [34.1–68.5]	*H* = 1.107	0.575
Tuberculosis severity					
Lung cavity count, Median [IQR]	3 [2–4]	3 [2–4]	3 [2–4]	*H* = 1.016	0.602
Sputum smear positivity (%), median [IQR]	53.4 [39.8–66.3]	56.6 [42.7–67.1]	54.1 [40.2–65.7]	*H* = 0.561	0.755
Comorbidities					
Diabetes mellitus, *n* (%)	112 (32.84%)	33 (38.82%)	69 (31.51%)	*χ*^2^ = 1.515	0.469
Hypoalbuminemia, *n* (%)	256 (75.07%)	67 (78.82%)	155 (70.78%)	*χ*^2^ = 2.418	0.299
Treatment regimens					
Rifampicin-containing, *n* (%)	265 (77.71%)	63 (74.12%)	184 (84.02%)	*χ*^2^ = 4.896	0.086
Isoniazid-containing, *n* (%)	320 (93.84%)	83 (97.65%)	207 (94.52%)	*χ*^2^ = 1.922	0.383
Pyrazinamide-containing, *n* (%)	250 (73.31%)	67 (78.82%)	173 (79.00%)	*χ*^2^ = 2.795	0.247
Ethambutol-containing, *n* (%)	287 (84.16%)	67 (78.82%)	185 (84.47%)	*χ*^2^ = 1.613	0.446
Nutrition protocol					
Tube type, *n* (%)				*χ*^2^ = 0.121	0.941
Nasogastric tube	159 (46.63%)	38 (44.71%)	100 (45.66%)		
Nasoenteric tube	182 (53.37%)	47 (55.29%)	119 (54.34%)		
Progressive mobilization, *n* (%)				*χ*^2^ = 0.759	0.684
Yes	150 (43.99%)	37 (43.53%)	104 (47.49%)		
No	191 (56.01%)	48 (56.47%)	115 (52.51%)		
Chinese herbal application, *n*(%)				*χ*^2^ = 4.570	0.102
Yes	180 (52.79%)	45 (52.94%)	135 (61.64%)		
No	161 (47.21%)	40 (47.06%)	84 (38.36%)		
Formula type, *n* (%)				*χ*^2^ = 2.570	0.277
Intact protein	214 (62.76%)	53 (62.35%)	123 (56.16%)		
Short-peptide	127 (37.24%)	32 (37.65%)	96 (43.84%)		
Calorie target (kcal), mean ± SD	1494.30 ± 196.57	1512.74 ± 184.38	1513.94 ± 192.50	*F* = 0.800	0.450
EN initiation time (h), median [IQR]	37.26 ± 8.54	38.95 ± 8.69	37.09 ± 8.47	*F* = 1.597	0.203
Outcome					
ENI, *n* (%)	136 (39.88%)	34 (40.00%)	87 (39.73%)	*χ*^2^ = 0.002	0.999

### 3.2 Performance testing of improved swarm intelligence algorithm

**Figure 2:**
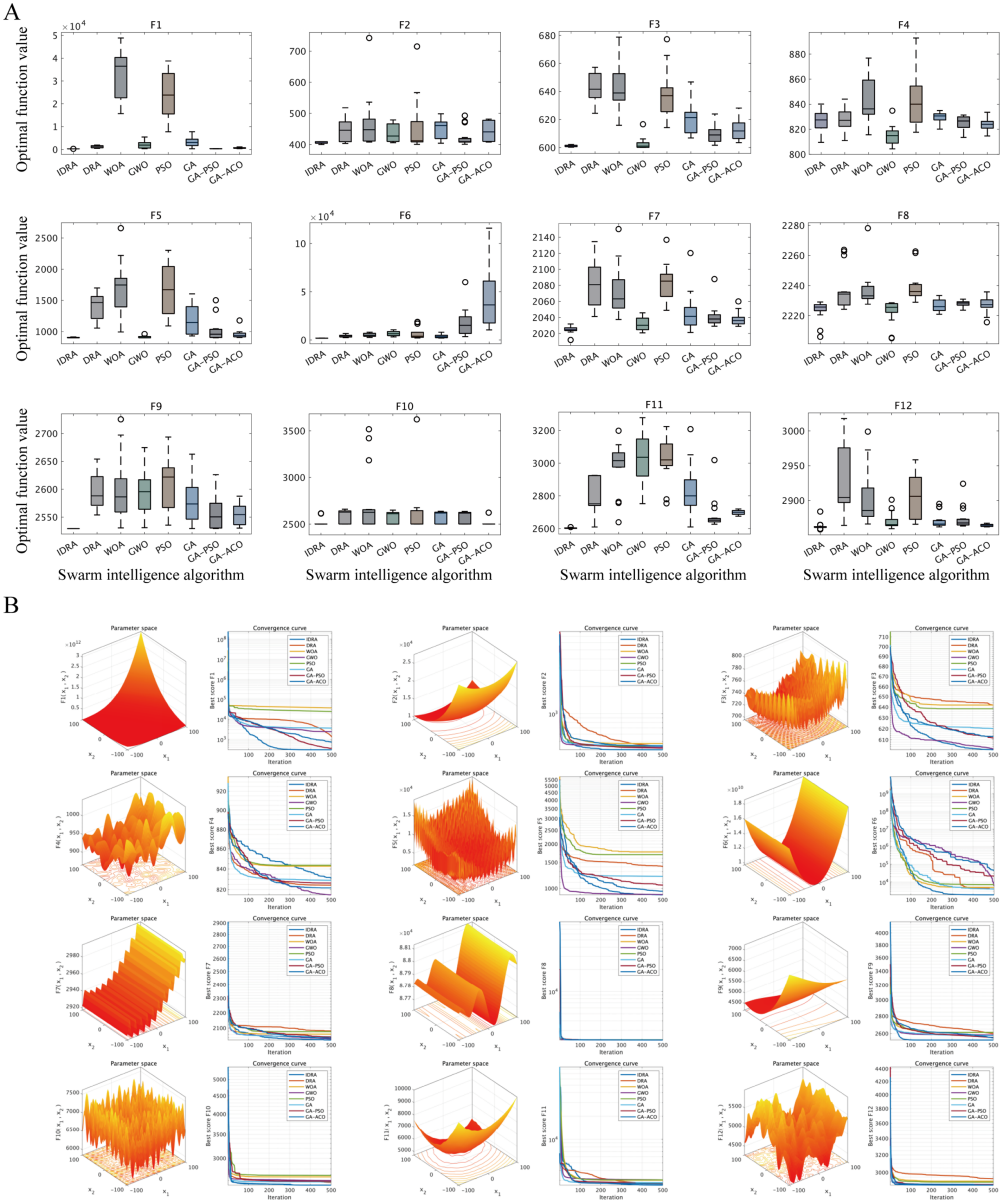
Comparison of optimization performance among swarm intelligence algorithms. **(A)** Box plots of optimization results after 30 independent runs of each algorithm on CEC2022 test functions, illustrating optimization stability and robustness across algorithms. **(B)** Convergence curves of algorithms during optimization, reflecting convergence speed and ability to avoid local optima.

IDRA demonstrated superior performance in most test functions, with significantly better stability than DRA and other comparative algorithms (Figure 2A). Convergence curve analysis further revealed that IDRA achieved faster convergence speed and exhibited the lowest risk of trapping in local optima during iterations (Figure 2B). These results robustly confirmed the substantial advantages of the IDRA algorithm in global optimization performance and convergence efficiency. The results of statistical tests are presented in Supplementary Material 2.

### 3.3 Model training performance

This study systematically evaluated the performance metrics of six machine learning models on the internal and external test sets. The results showed that the predictive performance of the AutoML model was significantly superior to that of the other models, achieving the highest AUC values of 0.910 (internal) and 0.880 (external) on both datasets. The XGBoost model ranked second (AUC 0.860/0.842). Regarding probability calibration, AutoML also demonstrated the best performance, with the lowest Brier score, indicating the highest predictive accuracy.

**Figure 3:**
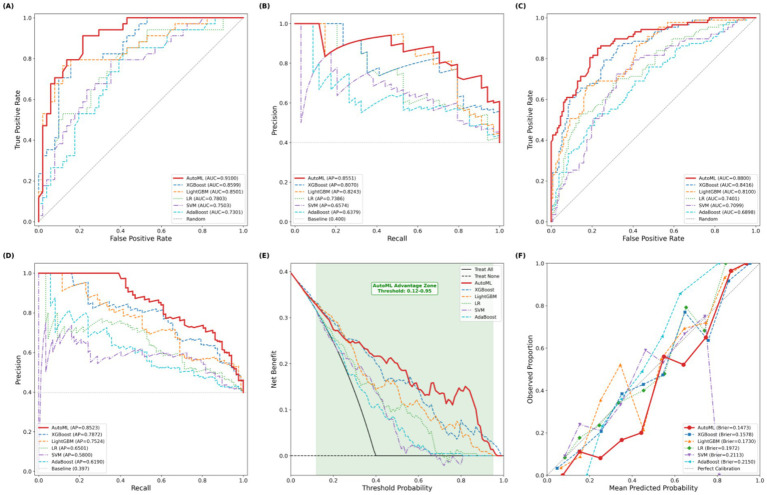
Comparative mapping of predictive model performance. **(A)** ROC curve on the internal test set; **(B)** PR curve on the internal test set; **(C)** ROC curve on the external test set; **(D)** PR curve on the external test set; **(E)** DCA curve on the external test set; **(F)** calibration curve on the external test set.

Among traditional machine learning models, Logistic Regression, Support Vector Machine, and AdaBoost exhibited relatively weaker performance, with their AUC values significantly lower than that of AutoML. Notably, the Logistic Regression model demonstrated the highest specificity but the lowest sensitivity, indicating that its predictive tendency was overly conservative. The results of the decision curve analysis indicated that the AutoML model provided the optimal net benefit across the clinically relevant threshold probability range (12 to 95%), and its predicted probabilities maintained good consistency with the actual event rates. Taken together, the AutoML ensemble model, through automated hyperparameter optimization and feature selection, fully integrated the strengths of multiple algorithms. This model not only possessed strong discriminative ability but also demonstrated excellent calibration performance, presenting significant advantages in actual clinical application scenarios. The AutoML was ultimately selected as the final model for this study. Its screened features were: Hypoalbuminemia, Anti-TB drug regimen (with/without rifampicin), Formula type (short peptide > intact protein), Age, BMI, EN initiation time, Tube type (nasoenteric > nasogastric), and Chinese herbal application (Figure 3 and Table 2).

**Table 2:** Performance evaluation metrics of predictive models.

Model	Dataset	AUC	Brier score	Accuracy	Sensitivity	Specificity	PPV	NPV
AutoML	Internal Test	0.9100	0.1311	0.8000	0.7941	0.8039	0.7297	0.8542
AutoML	External Test	0.8800	0.1473	0.8037	0.8506	0.7727	0.7115	0.8870
XGBoost	Internal Test	0.8599	0.1540	0.8118	0.7647	0.8431	0.7647	0.8431
XGBoost	External Test	0.8416	0.1578	0.7671	0.6552	0.8409	0.7308	0.7872
LightGBM	Internal Test	0.8501	0.1649	0.8118	0.7941	0.8235	0.7500	0.8571
LightGBM	External Test	0.8100	0.1730	0.7534	0.6207	0.8409	0.7200	0.7708
LR	Internal Test	0.7803	0.1945	0.7176	0.3235	0.9804	0.9167	0.6849
LR	External Test	0.7401	0.1972	0.7078	0.5402	0.8182	0.6620	0.7297
SVM	Internal Test	0.7503	0.2042	0.6706	0.6765	0.6667	0.5750	0.7556
SVM	External Test	0.7099	0.2113	0.6484	0.3793	0.8258	0.5893	0.6687
AdaBoost	Internal Test	0.7301	0.2149	0.6588	0.6471	0.6667	0.5641	0.7391
AdaBoost	External Test	0.6898	0.2150	0.6667	0.2759	0.9242	0.7059	0.6595

### 3.4 Analysis of key influencing factors

#### 3.4.1 LASSO

**Figure 4:**
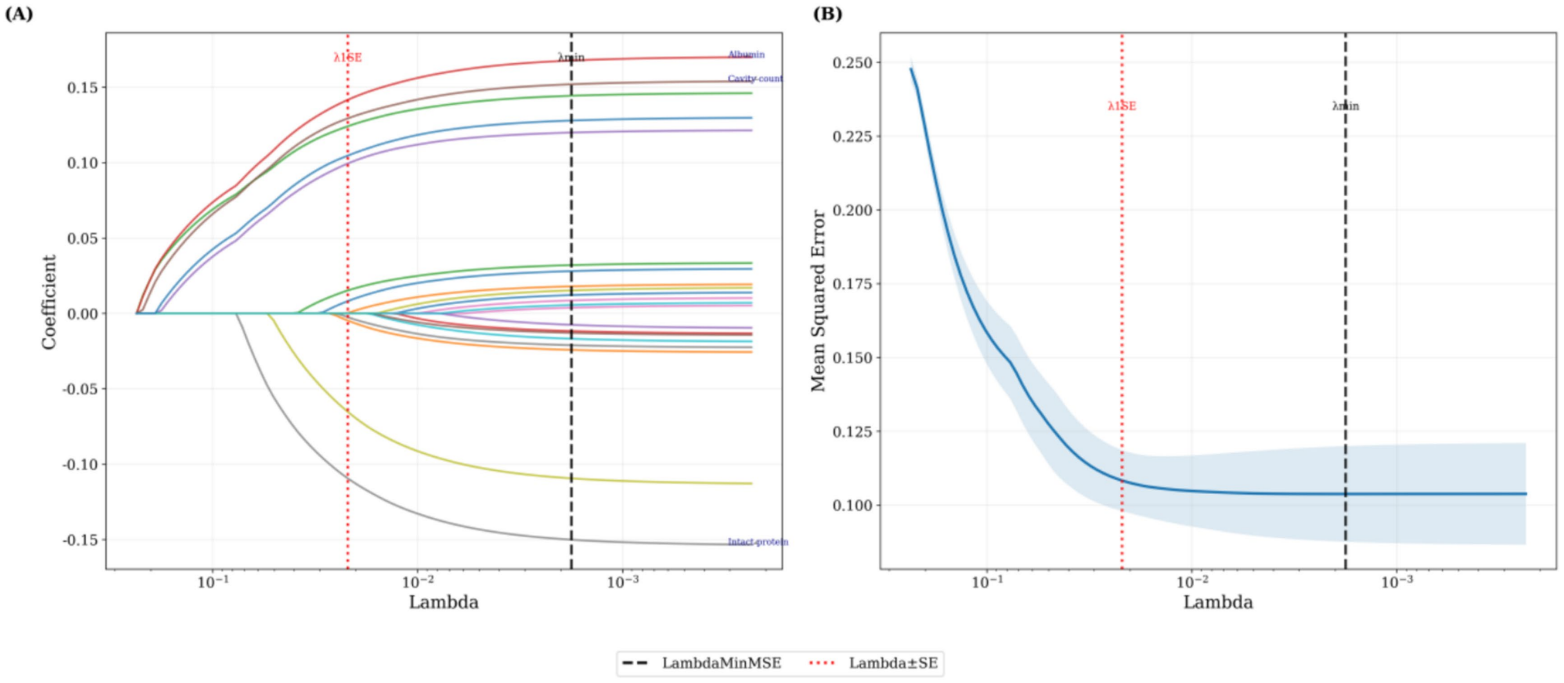
LASSO regression results. **(A)** LASSO trajectory plot; **(B)** LASSO cross-validation fit plot.

LASSO regression was employed for feature selection on the training set data (Figure 4) to verify the validity of the features screened by the AutoML model. LASSO selected variables within one standard error of the minimum MSE in the sparse model (Lambda1SE), screening out 10 variables: Hypoalbuminemia, Anti-TB drug regimen (with/without rifampicin), Anti-TB drug regimen (with/without isoniazid), CRP, Formula type, Age, BMI, EN initiation time, Progressive early mobilization, and Tube type, encompassing all features screened by AutoML.

#### 3.4.2 SHAP analysis

**Figure 5:**
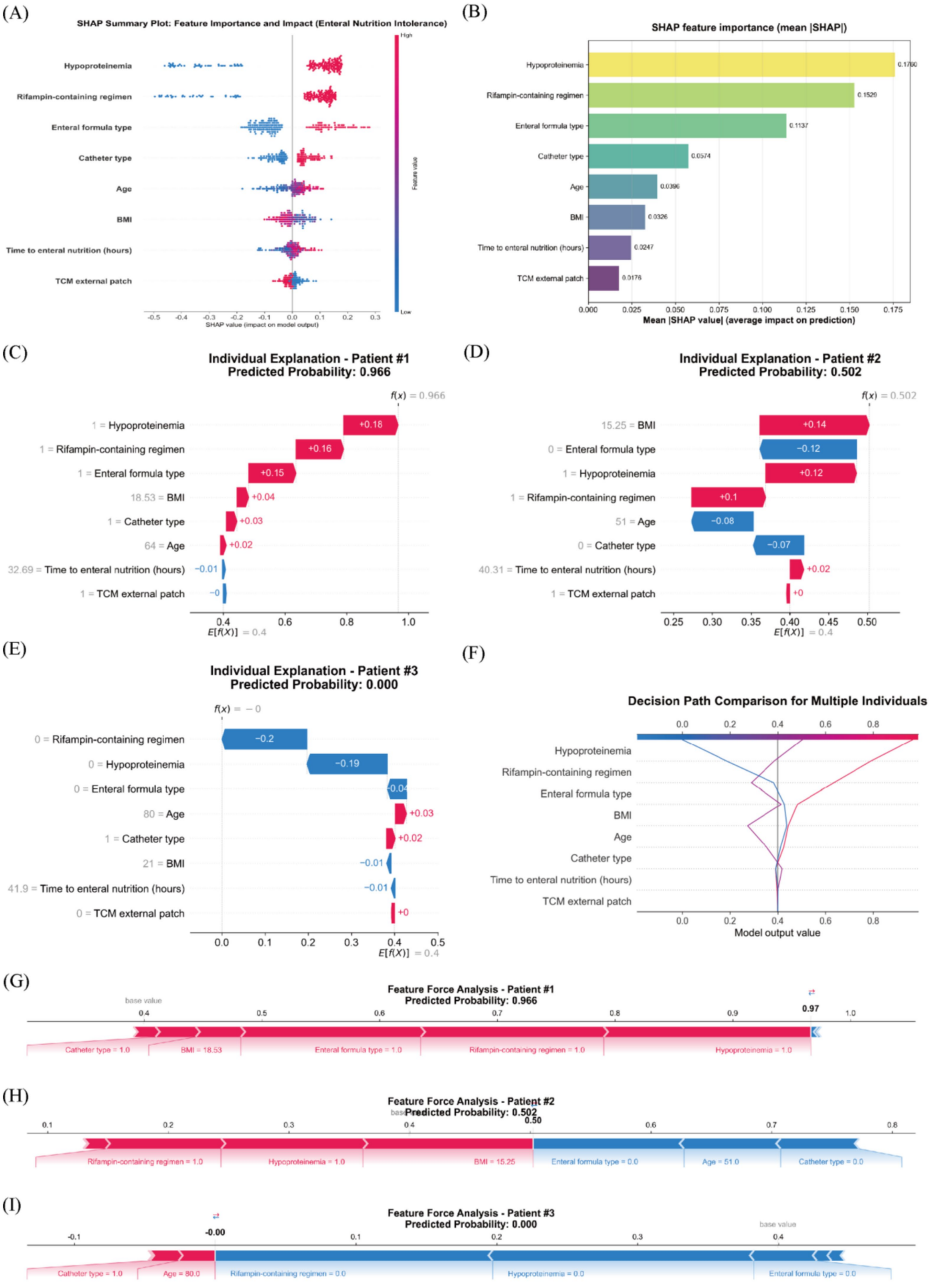
SHAP interpretability analysis. **(A)** Shapley summary plot. **(B)** Shapley feature importance plot. **(C–E)** Waterfall plots illustrating the cumulative contribution process of each feature to the individual patient’s prediction. The baseline value represents the model’s average prediction for all patients, while feature contributions show how each feature influences the final prediction (red indicates increased risk, blue indicates decreased risk). The sum of all feature contributions yields the final predicted value. **(F)** Decision plot comparing the decision paths of multiple patients, showing how different feature combinations lead to different prediction outcomes. The horizontal axis shows the predicted probability, the vertical axis lists the features, and the curved paths track the decision route from the baseline value to the final prediction. **(G–I)** Force plots intuitively demonstrate how each feature pushes the prediction toward a higher or lower risk direction. Red arrows indicate features pushing the prediction toward higher risk, blue arrows indicate features pushing it toward lower risk, and the arrow length represents the magnitude of the effect.

Based on a detailed interpretation of the Shapley Additive Explanations (SHAP) analysis results (Figures 5A,B), the study identified eight key predictive factors influencing intolerance. Their general direction of effect was (in descending order of importance): Hypoalbuminemia (presence increases risk) > Anti-TB regimen (rifampicin-containing regimen poses higher risk than non-containing) > Formula type (short peptide poses higher risk than intact protein) > Age (increased age poses higher risk) > BMI (lower BMI poses higher risk) > EN initiation time (later/longer initiation time poses higher risk) > Tube type (nasoenteric tube poses higher risk than nasogastric tube) > Chinese herbal application (non-use poses relatively higher risk). The relative weight analysis of these factors provided a theoretical basis for establishing an effective risk stratification model. By systematically comparing the characteristic combination patterns of patients at different risk levels (Figure 5F), the study revealed significant differential expression in clinical characteristics between high-risk and low-risk patients.

To demonstrate model interpretability at the individual level, one case each from high, medium, and low predicted probabilities of enteral nutrition intolerance was selected from the internal test set for analysis using waterfall plots, force plots, and individual SHAP contribution bar plots. A quantitative decomposition of the predicted probability by SHAP values was further reported, using the mean output of the test set as the baseline. The high-risk individual (Figures 5C,G) had a predicted probability of approximately 96.6%, presenting with hypoalbuminemia, a rifampicin-containing anti-tuberculosis regimen, short-peptide enteral nutrition, a BMI of 18.5 kg/m^2^, EN initiation at approximately 33 h, nasoenteric tube placement, and use of Chinese herbal application; these factors consistently and directionally pushed the prediction toward intolerance in the figure. Among them, the SHAP values for hypoalbuminemia, the rifampicin-containing regimen, and the short-peptide formula were +0.176, +0.155, and +0.153, respectively, jointly driving the high-risk output in the same direction, while BMI, nasoenteric tube, and age exhibited only modest positive contributions (+0.038, +0.034, +0.019), and EN initiation time and Chinese herbal application showed slightly negative contributions (−0.006, −0.003). This is consistent with the waterfall and force plot representations and aligns with clinical expectations for patients with higher nutritional risk. The medium-risk individual (Figures 5D,H) had a predicted probability of approximately 50.2%, also exhibiting hypoalbuminemia and a rifampicin-containing regimen, but with an intact protein formula, nasogastric tube, age of 51 years, low BMI (15.3 kg/m^2^), and relatively late EN initiation time (approximately 40 h). Positive and negative features coexisted: BMI (+0.141), hypoalbuminemia (+0.118), and the rifampicin-containing regimen (+0.096) elevated the risk, whereas the intact protein formula (−0.125), age (−0.080), and nasogastric tube (−0.065) markedly suppressed the prediction, causing the positive and negative effects to offset each other and placing the prediction in an uncertainty zone. The low-risk individual (Figures 5E,I) had a predicted probability close to 0; although aged 80 years, the model comprehensively judged an extremely low risk of intolerance due to the absence of hypoalbuminemia, a non-rifampicin-containing regimen, intact protein formula, and a BMI of 21.0 kg/m^2^, despite the use of a nasoenteric tube and non-use of Chinese herbal application. The corresponding SHAP analysis showed that the absence of hypoalbuminemia (−0.186), the non-rifampicin-containing regimen (−0.197), and the intact protein formula (−0.045) constituted the primary protective drivers, while advanced age (+0.026) and nasoenteric tube (+0.020) only produced mild positive effects that failed to offset these protective factors. The comparison of these three cases demonstrates the markedly differential outputs of the same model under different feature combinations, with per-feature SHAP values mutually corroborating the global summary results and completely illustrating the direction and magnitude of feature contributions across the high-to-low risk spectrum.

### 3.5 Clinical decision system construction

**Figure 6:**
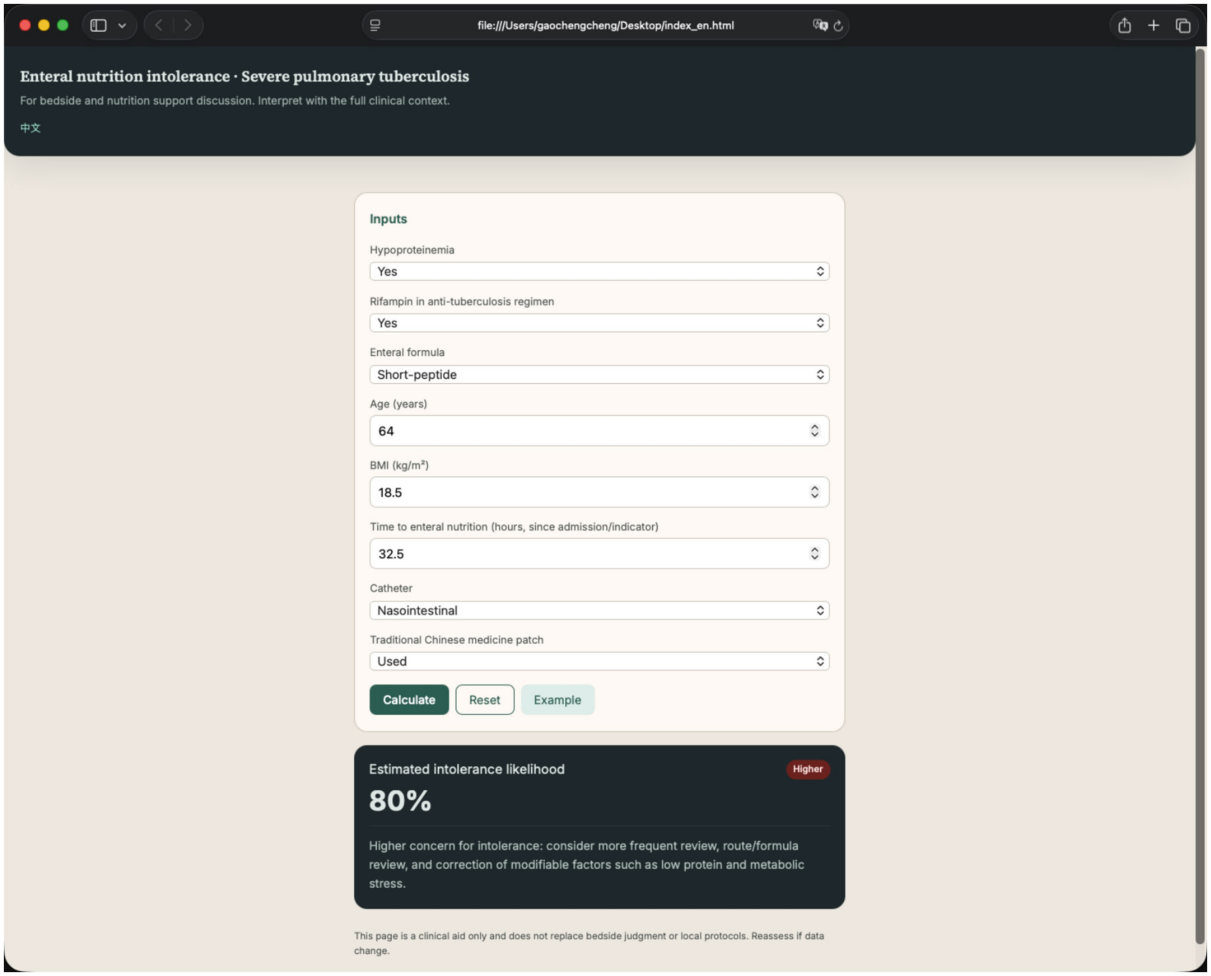
Software system demonstration.

The above research successfully identified key features influencing patient outcomes. However, in actual clinical application, the interplay of these features is complex, making it difficult to intuitively reveal a patient’s prognostic risk. Existing artificial intelligence methods have a high threshold for dissemination and application, requiring clinical staff to possess substantial programming skills and extensive literature knowledge, making them challenging to promote in many hospitals. To address this issue, this study innovatively constructed a practical visualization system. This system was built upon the selected key features, possessing the application advantages of intuitiveness, convenience, and practicality. In the application process of the visualization system, users only need to input the specific values of nine key features into the “Feature Input” column, and the system automatically calculates the patient’s risk of enteral nutrition intolerance and provides clinical recommendations, as shown in Figure 6.

## 4 Discussion

Tuberculosis is a chronic consumptive infectious disease whose occurrence, development, and prognosis are closely associated with nutritional status. Annually, approximately 100,000–300,000 tuberculosis patients require intensive care treatment, with mortality exceeding 50% (17). Clinically, gastrointestinal function improvement in critically ill patients involves pharmacological and non-pharmacological interventions; while drug interventions are effective, they carry adverse effects. As a key non-pharmacological intervention, enteral nutrition (EN) provides nutritional support, enhances gastrointestinal function, and reduces complications in severe pulmonary tuberculosis (PTB) patients (18, 19). This study constructed a model to identify influencing factors and screen pivotal features, offering evidence for clinical decision-making.

In the AutoML framework for constructing predictive models, the selection of an appropriate underlying optimization algorithm is crucial for determining the quality of feature selection and hyperparameter tuning. After comparing the applicability of various classical metaheuristic algorithms to mixed-integer optimization problems, this study adopted the Divine Religions Algorithm (DRA) as the foundational optimization core. Compared with common approaches such as GA, ACO, DRA demonstrates superior structural compatibility with this task: GA’s search capability heavily relies on the elaborate design of crossover and mutation operators, and when faced with the simultaneous optimization of high-dimensional feature combinations and continuous hyperparameters, it is prone to converge prematurely to local optima due to rapid loss of population diversity; ACO relies on a pheromone matrix-guided path construction mechanism, which struggles to naturally represent a non-routing joint space that simultaneously includes discrete feature decisions and continuous parameter values. By simulating the two coexisting mechanisms of “faith conversion” for macro-exploration and “missionary work” for local deep exploitation within religious systems, DRA inherently builds a dynamic balance between exploration and exploitation in its algorithmic structure, facilitating sustained search vitality in multimodal, highly interactive clinical data optimization landscapes. However, the standard DRA is still limited by the arbitrariness of population initialization and stagnation in local optima due to elite individual assimilation in the later stages of search. To address this, this study proposes an Improved Divine Religions Algorithm (IDRA) based on dimension-wise Gaussian mutation and chaos, whose improvements are not merely aimed at numerical superiority on benchmark functions, but are grounded in deep optimization theory. First, IDRA uses Cubic chaotic mapping instead of a pseudorandom number generator to initialize the population: the deterministic sequence generated by the cubic map possesses better traversal uniformity and low sensitivity to initial conditions, enabling the initial individuals to more comprehensively occupy the search space, thereby significantly reducing the risk of systematically missing key biomarker combinations due to poor initial distribution, which is particularly critical for clinical data with complex nonlinear associations among features and high noise. Second, for the optimal individual in each generation, IDRA implements dimension-wise Gaussian mutation: the algorithm retains the overall structure of the elite individual, only applies a small perturbation following a Gaussian distribution to each dimension in turn, and adopts the change only if the fitness improves. This dimension-wise conservative fine-tuning mimics the natural mechanism of progressive evolution in which an elite individual, having stabilized its acquired knowledge backbone, explores through single-factor independent trials. This enables the algorithm to protect the important feature dimensions already discovered while continuously exploring adjacent optimization directions, thus significantly enhancing the ability to escape pseudo-local optima and conduct high-precision supplementary searches. Combined with systematic test results on the CEC2022 benchmark functions, IDRA demonstrated faster convergence speed and a lower risk of falling into local optima, validating the effectiveness of the aforementioned theoretical mechanisms. This provides a computationally sound and stable optimization engine for the automated selection of highly robust feature subsets in the prediction of enteral nutrition intolerance in severe pulmonary tuberculosis patients in this study.

The correlation between identified factors and enteral nutrition intolerance in severe PTB patients represents a core finding of this study. Through dual-dimensional validation via LASSO regression and SHAP interpretability analysis, we confirmed critical predictors, including hypoalbuminemia, anti-TB drug regimens (rifampicin-containing or not), formula type (short peptide vs. intact protein), age, BMI, EN initiation time, tube type (nasoenteric vs. nasogastric), and Chinese herbal application. These factors demonstrated strong associations with EN gastrointestinal outcomes. Gastrointestinal mucosal repair and digestive enzyme synthesis rely on adequate protein; hypoalbuminemia induces mucosal atrophy, barrier impairment, and insufficient enzyme activity, leading to malabsorption and elevated risks of abdominal distention and diarrhea (20). Rifampicin, as a hepatocyte enzyme inducer, accelerates digestive enzyme metabolism and reduces their activity. It also irritates the gastrointestinal mucosa, diminishing EN tolerance (21, 22). Short-peptide formulas are directly absorbable and suitable for patients with compromised GI function, whereas intact proteins may cause discomfort due to incomplete digestion. Older age and low BMI populations exhibit more pronounced GI functional decline. Early EN initiation maintains physiological stimulation, preserving GI motility and barrier function (23). Progressive mobilization enhances GI peristalsis and reduces intolerance symptoms. Nasoenteric tubes bypass gastric digestion in individuals with impaired gastric motility. Chinese herbal acupoint application, a Traditional Chinese Medicine intervention, stimulates acupoints transdermally, enhancing gastrointestinal motility and improving gastric emptying, thereby reducing ENI risk (24, 25). The clinical relevance of these factors was further corroborated through SHAP’s global interpretation. Specifically, SHAP quantifies each feature’s influence weight on “enteral nutrition intolerance” by computing mean absolute SHAP values across predictive outcomes, perfectly addressing clinical demands for validating “influence factor association strength” (26, 27). Additionally, SHAP employs game-theoretic Shapley values to ensure mathematically unbiased contribution attribution, avoiding spurious correlation interpretations. Visualization via SHAP summary plots and dependence diagrams translates complex model decisions into clinically intuitive graphics, enabling researchers to rapidly discern factor-risk relationships and directly support clinical conclusions.

To further demonstrate the model’s individualized interpretability, representative cases spanning high, medium, and low predicted probabilities of enteral nutrition intolerance (ENI) were selected from the internal test set for detailed analysis using SHAP waterfall plots, force plots, and individual contribution bar plots. The high-risk individual exhibited a predicted probability of approximately 96.6%, characterized by hypoalbuminemia, a rifampicin-containing anti-tuberculosis regimen, short-peptide enteral nutrition, a BMI of 18.5 kg/m^2^, EN initiation at approximately 33 h, nasoenteric tube placement, and use of Chinese herbal application; these factors collectively and consistently drove the prediction toward the “intolerance” outcome, aligning with clinical expectations for patients presenting with higher nutritional risk profiles. The medium-risk individual had a predicted probability of approximately 50.2%, also presenting with hypoalbuminemia and a rifampicin-containing regimen, but with an intact protein formula, nasogastric tube, age of 51 years, notably low BMI (15.3 kg/m^2^), and relatively late EN initiation time (approximately 40 h); in this case, risk-increasing and risk-decreasing features coexisted, placing the prediction within an uncertain zone that reflects the clinical dilemma frequently encountered in such borderline patients. The low-risk individual yielded a predicted probability close to 0; although aged 80 years, the model comprehensively evaluated the ENI risk as extremely low due to the absence of hypoalbuminemia, a non-rifampicin-containing regimen, intact protein formula, and a BMI of 21.0 kg/m^2^, despite the use of a nasoenteric tube and non-use of Chinese herbal application. The juxtaposition of these three cases vividly illustrates the differential outputs of the AutoML model under varying feature combinations, providing confirmation that is mutually supportive with the global SHAP results regarding the effect direction and relative importance of the eight key predictors. Specifically, the comparison reinforces that hypoalbuminemia and rifampicin-containing regimens are the predominant risk drivers, while their absence, in conjunction with a normal BMI, can substantially mitigate the predictive risk even in the presence of other potentially unfavorable conditions. This granular, case-based interpretability offers clinicians a transparent window into the model’s decision-making logic, fostering trust and laying a foundation for translating model predictions into personalized, actionable clinical insights at the bedside.

However, it is important to interpret these associative findings with caution. The SHAP analysis revealed that nasoenteric tube placement and short-peptide formula were associated with higher predicted ENI risk compared to nasogastric tube placement and intact protein formula, respectively—findings that appear counterintuitive given that nasoenteric tubes are designed to bypass gastric dysfunction and short-peptide formulas are specifically formulated for patients with compromised gastrointestinal function. We believe these associations most likely reflect confounding by indication rather than a causal relationship: in clinical practice, nasoenteric tubes and short-peptide formulas are preferentially selected for patients who already present with known or suspected gastrointestinal dysfunction, i.e., those at inherently higher risk of intolerance. Indeed, as described in our individual case analyses, the SHAP contributions of nasoenteric tube and short-peptide formula in the high-risk individual were observed in the context of multiple concomitant risk factors, including hypoalbuminemia and a rifampicin-containing regimen, which exerted substantially larger effects; conversely, in the low-risk individual, nasoenteric tube placement contributed only a mild positive effect that was readily offset by protective features such as the absence of hypoalbuminemia and a non-rifampicin-containing regimen. These patterns support the interpretation that the observed associations are driven by underlying patient severity rather than by the interventions themselves. Nevertheless, these findings highlight the clinical reality that patients receiving nasoenteric tubes and short-peptide formulas represent a higher-risk subpopulation warranting intensified monitoring, and they underscore the need for prospective studies to disentangle treatment effects from selection effects.

Although this study achieved breakthroughs in building an enteral nutrition intolerance prediction model and clinical decision system for severe PTB, several limitations warrant further investigation. Data were derived from an exploratory cohort of 426 patients at two centers, restricting sample representativeness and diversity and potentially compromising external model validation—necessitating future multi-center validation. Despite encompassing TB-specific indicators like anti-TB regimens and lung cavity count, inadequate standardization in electronic health records resulted in missing key variables (e.g., dynamic serum TB antibody titers). Manual data entry also introduced temporal variable errors (e.g., EN initiation time), whose heterogeneity directly affected model generalizability. In model construction, while the AutoML ensemble approach effectively integrated multiple algorithms, its reliance on static feature selection may still limit dynamic clinical data processing. For instance, the current model predicts ENI risk at a single time point, potentially overlooking temporal pathophysiological mechanisms like progressive intestinal dysbiosis subtypes. The current clinical decision system, while featuring a user-friendly visualization interface, still lacks direct integration with hospital information systems (HIS), requiring manual data entry that increases clinical workload and hinders widespread adoption in primary hospitals.

Addressing these challenges requires innovative solutions across multiple dimensions. Establishing a multi-center severe PTB research collaborative network using standardized data collection protocols and federated learning could enhance prediction generalization without compromising data privacy. Future models must overcome static prediction constraints by developing spatiotemporal prediction systems: integrating AutoML-based feature optimization with LSTM neural networks for temporal feature modeling, enabling dynamic risk assessment throughout the EN process. Neuro-symbolic AI techniques that convert SHAP values into clinically executable rules will augment trustworthiness. Future practical applications may demand hierarchical decision ecologies: upgrading the current visualization system to enable real-time bedside risk assessment via mobile devices, automatic personalized intervention recommendations for high-risk patients, and integration with hospital EHR systems for seamless data flow and closed-loop management.

In conclusion, this study systematically constructed a comprehensive framework spanning “data processing–model optimization–clinical validation.” Its core value extends beyond outperforming traditional predictive efficacy—it pioneers an interpretable, operable, and efficient precision risk assessment prototype, offering novel insights into early prediction and prevention of enteral nutrition intolerance in severe PTB patients.

## Data Availability

The raw data supporting the conclusions of this article will be made available by the authors, without undue reservation.
