# Supplementary material for: An interpretable AutoML-based prediction model for enteral nutrition intolerance in severe pulmonary tuberculosis patients and development of a clinical decision system
Source: Front Nutr. 2026 Jun 22;13:1816436. doi: 10.3389/fnut.2026.1816436 (PMC13333408; doi:10.3389/fnut.2026.1816436)
Supplement: Supplementary file 1 [file Table_1.DOCX]

**Statistical Tests of Swarm Intelligence Algorithms on CEC2022 Unimodal Benchmark Functions**

**1. Overall Validation: Friedman and Iman–Davenport Tests**

On the 12 unimodal benchmark functions from the CEC2022 test set, a non-parametric Friedman test was conducted using the test functions as blocks and the eight algorithms as treatments, with the minimized objective function value (error, smaller is better) as the dependent variable. The Friedman statistic was χ² = 55.58 (number of algorithms k = 8, number of blocks N = 12, degrees of freedom = 7), yielding p = 1.14 × 10⁻⁹, indicating statistically significant performance differences among the eight algorithms when jointly considering all 12 test functions. After applying the Iman–Davenport correction to the Friedman test, F = 21.52 (df₁ = 7, df₂ = 77), p = 8.88 × 10⁻¹⁶, the conclusion remained highly significant.

**2. Post-Hoc Pairwise Comparisons: IDRA versus the Seven Comparison Algorithms**

Based on the overall test confirming differences among the eight algorithms, IDRA was set as the control algorithm. For each test function, the mean error of each algorithm over 30 independent runs was first calculated, and then paired differences were computed as dⱼ = (IDRA mean error)ⱼ − (comparison algorithm mean error)ⱼ, yielding 12 paired differences across the test functions. A two-sided Wilcoxon signed-rank test was applied to d₁–d₁₂, and Holm sequential correction was performed for all seven pairwise comparisons (IDRA vs. DRA, WOA, GWO, PSO, GA, GA-PSO, and GA-ACO). After correction, all seven comparisons were significant at α = 0.05 (all pHolm < 0.05), demonstrating that none of the seven comparison algorithms outperformed IDRA in terms of the comprehensive paired differences across the 12 functions. See Table S1.

**Table S1. Post-Hoc Pairwise Comparisons between IDRA and the Seven Comparison Algorithms**

| Comparison | Number of Blocks (Test Functions N) | pHolm | Reject Null Hypothesis at Two-Sided α = 0.05? |
| --- | --- | --- | --- |
| IDRA vs DRA | 12 | 1.95×10^-3^ | Yes |
| IDRA vs WOA | 12 | 1.95×10^-3^ | Yes |
| IDRA vs GWO | 12 | 6.84×10^-3^ | Yes |
| IDRA vs PSO | 12 | 1.95×10^-3^ | Yes |
| IDRA vs GA | 12 | 1.95×10^-3^ | Yes |
| IDRA vs GA-PSO | 12 | 2.93×10^-3^ | Yes |
| IDRA vs GA-ACO | 12 | 6.84×10^-3^ | Yes |

**3. Win/Tie/Loss Counts Based on Per-Function Two-Sample Tests**

Within each individual test function, a two-independent-sample Mann–Whitney U test was performed on the errors from the 30 independent runs of each algorithm pair, covering all seven comparison algorithms other than IDRA. When p < 0.05, the algorithm with the lower median error was deemed better; when p ≥ 0.05, the result was considered a tie. A “win” denotes that the test was significant at the nominal α = 0.05 and IDRA exhibited a lower median error; a “loss” denotes that the comparison algorithm was significantly better. The aggregated win/tie/loss frequencies across the 12 unimodal functions were as follows: vs. DRA 11/1/0, vs. WOA 12/0/0, vs. GWO 7/4/1, vs. PSO 12/0/0, vs. GA 11/1/0, vs. GA-PSO 11/1/0, vs. GA-ACO 10/2/0.
